# Minimally invasive surgery using intraoperative electron-beam radiotherapy for the treatment of soft tissue sarcoma of the extremities with tendon involvement

**DOI:** 10.1186/s12957-016-0968-4

**Published:** 2016-08-12

**Authors:** Akihiko Matsumine, Masaya Tsujii, Tomoki Nakamura, Kunihiro Asanuma, Takao Matsubara, Takuya Kakimoto, Yuki Yada, Akinori Takada, Noriko Ii, Yoshihito Nomoto, Akihiro Sudo

**Affiliations:** 1 Department of Orthopaedic Surgery, Mie University Graduate School of Medicine, 2-174 Edobashi, Tsu City, Mie 514-8507 Japan; 2 Department of Radiology, Mie University School of Medicine, 2-174 Edobashi, Tsu city, Mie 514-8507 Japan

**Keywords:** Minimally invasive surgery, Intraoperative electron-beam radiotherapy (IOERT), Soft tissue sarcoma, Tendon involvement, Clinical outcome

## Abstract

**Background:**

When a soft tissue sarcoma (STS) is located at the distal part of an extremity and involves the tendon, a wide excision usually causes severe functional disability. We therefore developed a minimally invasive surgical technique using intraoperative electron-beam radiotherapy (IOERT) to reduce the incidence of post-operative functional disability in patients with peri-/intra-tendinous STS. We assessed the clinical outcomes of the novel minimally invasive surgery.

**Methods:**

The study population included five patients who received treatment for distal extremity STSs. After elevating the tumor mass, including the tendon and nerve from the tumor bed with a wide margin, a lead board was inserted beneath the tumor mass to shield the normal tissue. IOERT (25–50 Gy) was then applied, and the tumor excised with care taken to maintain the continuity of the tendon.

**Results:**

In a desmoid patient, local recurrence was observed outside the irradiated field. No cases of neuropathy or bone necrosis were observed. The mean limb function score was excellent in all patients. None of the high-grade sarcoma patients had local recurrence or distant metastasis.

**Conclusions:**

Although the current study is only a pilot study with a small number of patients, it shows that this minimally invasive procedure has the potential to become a standard treatment option for selected patients.

**Trial registration:**

H17-250 (registered 2 November 2005) and H25-250 (modified from H17-250, registered 5 December 2013)

## Background

Although the recent progression of multidisciplinary treatments, such as chemotherapy agents, molecular target drugs, and ion beam radiotherapy, have enabled the application of various therapeutic options in the treatment of soft tissue sarcoma (STS), the resection of the tumor with a wide margin is the standard and most reliable treatment for patients with extremity STS [1, 2].

The surgical resection of STS with a wide margin sometimes causes functional disability of the affected limb, because a wide resection requires the excision of adjacent structures, including the bones, nerves, arteries, veins, and tendons [1–4]. The wide excision of even small tumors will usually result in severe functional disability if they are peri-/intra-tendinously located at the distal part of an extremity [1, 3–5]. The active motion of the finger and wrist may be impaired in patients’ forearm tumors. If the tumor is located at the distal leg, the active motion of the toe and ankle may be impaired.

Surgeons have attempted to reduce the degree of functional disability after the resection of peri-/intra-tendinous STSs by various reconstruction methods, including tendon transfers [5, 6], autologous tendon grafts [7–10], allogenic tendon grafts [11], and functional muscle transfers [3, 4, 6, 7, 12]. However, these procedures require the muscle to act as a power source, and muscle function may disappear after a wide excision [4, 7, 12]. Tendon transfer causes severe soft tissue adhesion due to the sub-optimal condition of the tissue bed after resection [13]. Although a tendon graft may be a treatment option, the harvest of the tendon results in surgical stress at the donor site [7, 11]. The clinical outcome of the reconstruction of the soft tissue defect should be measured not only by stable wound coverage but also by preservation of a patient’s limb function [6]. However, the impact of reconstructive surgery has been limited [12].

External-beam radiation therapy (EBRT) given before or after surgery is the standard treatment, especially in high-grade (G2–3), deep, and less than 5-cm lesions [2]. Preoperative EBRT is associated with lower rates of late toxicity and may allow a lower radiation dose, because the intact vascular supply renders a tumor more sensitive to RT. Post-operative EBRT is typically administered at 60–68 Gy over 6 to 7 weeks. Advantages of post-operative EBRT include the availability to conduct a full pathological review of the tumor prior to making a decision about adjuvant therapy. However, the most critical problem associated with EBRT is that it can cause a number of common, well-described side effects, such as muscle fibrosis, severe neuropathy, joint contracture, vascular obstruction, and lymphatic edema [14, 15]. These adverse effects of EBRT sometimes compromise the quality of life of patients.

Intraoperative radiotherapy (IORT) was first performed by Abe et al. at Kyoto University in the early 1960s [16]. IORT is designed to provide a large radiation dose to a target tissue while sparing normal tissues. Intraoperative electron-beam radiotherapy (IOERT) refers to the application of electron-beam radiation in this procedure. The therapeutic advantage is that IOERT can deliver a highly effective radiation dose to a specific target, while the dose to healthy structures can be limited by surgical displacement or shielding [17]. There is a large amount of published work on the use of IOERT in combination with surgery with/without EBRT in the treatment of soft tissue sarcomas of the retroperitoneum or extremities [14, 18–27]. The typical IOERT doses range from 10 to 20 Gy. Doses of 10–12.5 Gy are often used for patients with microscopic residual or close margins. However, for patients with gross residual disease, higher doses of up to 20 Gy are used. Recently, the combination of IOERT and EBRT has been recommended and many institutes perform IOERT (15 to 20 Gy) followed by EBRT (35 to 50 Gy) after surgery [23]. The 5-year local control rates in patients with retroperitoneal STSs and extremity STSs were reported to be 40–65 % [22, 24, 25] and 63–91 % [14, 17–21, 27, 28], respectively.

Although conventional IOERT has been used to achieve limb preservation without local recurrence, the improvement of limb function has not usually been considered in the assessment of treatment outcomes. We therefore developed a minimally invasive surgical technique using IOERT to reduce the incidence of post-operative functional disability in patients with peri-/intra-tendinous STS.

This surgical procedure consists of the four following steps: 1) the elevation of the tumor mass including the tendon and nerve from the tumor bed with a wide margin, 2) IOERT after the insertion of a lead board beneath the tumor mass to shield the normal tissue, 3) tumor excision maintaining the continuity of the tendon, and if necessary, 4) reconstruction of the soft tissue defect using a vascularized/local musculocutaneous flap. The purpose of the present study is to clarify the clinical outcomes of this novel surgical technique.

## Methods

### Patients

Five patients with peri-/intra-tendinous STSs of the distal extremities underwent surgical treatment in combination with IOERT (Table 1). All of the patients were female. The mean age of the patients was 32 years (range 14–62 years). The mean follow-up period was 50 months (range 27–67 months). The tumors were located intra-tendinously in three cases and peri-tendinously with adhesion to the tendon in two cases. No patients had distant metastasis. The mean tumor size was 3.1 cm (range 0.7–4 cm). The histological diagnoses were clear cell sarcomas (*n* = 2) and synovial sarcoma (*n* = 1), desmoid (*n* = 1), and myxofibrosarcoma (*n* = 1). The involved tendons were flexor/extensor of the distal forearm (*n* = 2), the calcaneal tendons (*n* = 2), and the tibialis posterior tendon (*n* = 1). Two of five operations were additional excisions after intra-lesional resection at another hospital. One desmoid patient was a recurrent case who had undergone three previous surgeries. Adjuvant chemotherapy was performed in cases 2 and 3. The study was performed in accordance with the ethical standards of the institutional responsible committee on human experimentation and with the Helsinki Declaration of 1975, as revised in 1983. All patients provided written informed consent before enrolment in the study.

**Table 1:** The clinical details of the patients

No.	Age	Gender	Diagnosis	Location	Condition at initial treatment	Involved tendon	Location	Tumor size (cm)	Histologic grade	Metastasis	Chemotherapy
1	14	f	Desmoid	Forearm	Recurrence (three times)	FDS, FDP	Intra-tendinous	4	Intermediate	No	No
2	21	f	Clear cell sarcoma	Leg	Primary	Calcaneal tendon	Intra-tendinous	4	High grade	No	Yes
3	28	f	Synovial sarcoma	Leg	Inadequate resection at initial hosp	Calcaneal tendon	Peri-tendinous	0.7	High grade	No	No
4	36	f	Clear cell sarcoma	Ankle	Primary	TP	Intra-tendinous	3	High grade	No	Yes
5	62	f	Myxofibrosarcoma	Forearm	Inadequate resection at initial hosp.	EDC	Peri-tendinous	4	High grade	No	No

*FDS* flexor digitorum superficialis, *FDP* flexor digitorum profundus, *TP* tibialis posterior, *EDC* extensor digitorum communis

### Surgical procedures, IOERT, and post-operative management

**Fig. 1:**
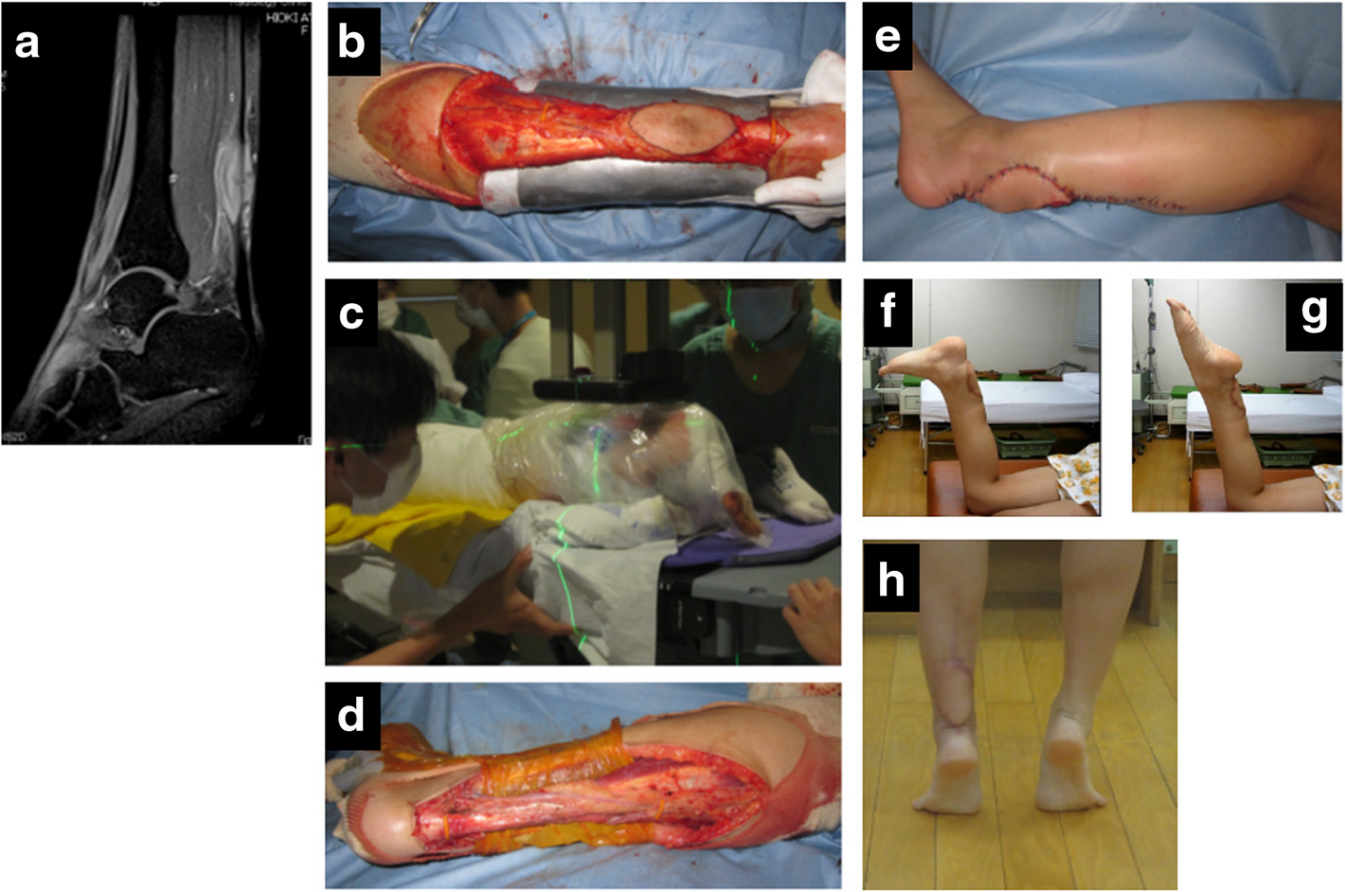
Surgical procedure and post-operative limb function in case 2, (**a**) T2 weighted-MR image showing clear cell sarcoma located intra-tendinously at calcaneal tendon. **b** After the elevation of the tumor mass, including the tendon and nerve from the tumor bed, a 6-mm-thick lead boards were inserted beneath the tumor mass to shield the normal tissue (**c**). The patients were transferred to the radiotherapy department after the affected limbs were wrapped in sterilized plastic bags, and 35–50 Gy of 12 MeV electrons were delivered. **d** After irradiation, the patients were returned to the operation room, and underwent the marginal/intra-lesional resection of the tumor, preserving the tendon and nerve. **e** The soft tissue defect was then reconstructed using a free musculocutaneous flap. The photographs at the final follow-up time showing the normal ankle movement (**f** and **g**) and ability of stand on toe (**h**)

The lesion was isolated and separated from the normal tissues in the axial plane with a wide surgical margin, maintaining the continuity of proximal and distal normal tissue. After the elevation of the tumor mass, including the tendon and nerve, from the tumor bed, 6-mm-thick lead boards, which were covered with a sterile drape, were inserted beneath the tumor mass to shield the normal tissue (Fig. 1a). The wet gauze was used as a layer of low density material to protect the braking X-ray. The patients were transferred to the radiotherapy department after the affected limbs were wrapped in sterilized plastic bags (Fig. 1b), and 25–50 Gy of 12 MeV electrons were delivered by a linear accelerator (Novalis TX, Brain LAB, Feldkirchen, Germany). Using the linear-quadratic (LQ) model (assuming an alpha/beta of three for the normal soft tissue surrounding the tumor), a single dose of 25, 35, and 50 Gy corresponded to an equivalent dose in 2-Gy fractions (EQD2) of 140, 266, and 530 Gy, respectively [29]. An appropriately sized cone was used to obtain a 3-cm longitudinal safety-margin for the irradiation field. After irradiation, the patients were returned to the operation room, and underwent the marginal/intra-lesional resection of the tumor, preserving the tendon and nerve (Fig. 1c) under general anesthesia. When the muscle was included in the irradiation field, the muscle was resected as much as possible. If necessary, the soft tissue defect was then reconstructed using a pedicled or free musculocutaneous flap (Fig. 1d).

After the operation, all of the patients were kept immobilized with the extremity in a bulky dressing for the first 12 h. Thereafter, the patients started a gentle active range of motion (ROM) exercise. Extensive rehabilitation was started after confirming the survival of the flap on post-operative day 10. Full weight bearing was permitted at 3 weeks after surgery in cases 2, 3, and 4.

### Assessment

The complications, local recurrence, oncological result, and functional outcome were evaluated. The functional assessments were performed using the Musculoskeletal Tumor Society (MSTS) scoring system [30]. The Radiation Therapy Oncology Group (RTOG) criteria were used to analyze the toxicity [15].

## Results

In all of the cases, an appropriately sized cone was used to obtain a 2-cm longitudinal safety-margin for the irradiation field. After irradiation, the tumor was surgically resected with the marginal margin (case 4) or intra-lesional margin (cases 1, 2, 3, and 5) (Table 2). The median follow-up period was 56 months (range 27–67 months).

**Table 2:** Clinical outcomes of the patients

No.	Radiation doses	Cone size (cm)	Size of irradiated lesion (cm)	Margin status	Reconstruction of the skin defect	Follow-up period (months)	Oncologic result	Limb function (ISOLS)	Complication (RTOG grade)
1	25 Gy	15	8 × 3	Positive	No	63	AWD	77	rec., restricted ROM of the wrist (grade 1)
2	35 Gy	15	12 × 4	Positive	Free vascularized flap	67	CDF	100	None
3	35 Gy	10	8 × 4	Positive	Free vascularized flap	56	CDF	100	Partial flap necrosis
4	50 Gy	10	8 × 4	Negative	Pedicle flap	36	CDF	100	None
5	35 Gy	15	10 × 4	Positive	Free vascularized flap	27	CDF	97	Restricted extension of the wrist joint

Margin status: surgical margin when tumor was resected

*AWD* alive with disease, *CDF* continuous disease free, *rec.* recurrence, *ROM* range of motion, *ISOLS* Scoring System of International Society of Limb Salvage, *RTOG* radiation therapy oncology group

For one desmoid patient, the entire operation was performed under general anesthesia (case 1). A patient whose tumor was located at the forearm (case 5) started the procedure under a supraclavicular brachial plexus block, and the procedure was started under lumbar anesthesia in three patients with leg tumors (cases 2, 3, and 4). However, after the patients returned to the operation room following the IOERT, the tumor resection and reconstruction of the soft tissue defect were performed under general anesthesia. A free vascularized latissimus dorsi flap was used to reconstruct the soft tissue defect in cases 2, 3 and 5; a localized pedicle musculocutaneous flap was used in case 4. A patient with synovial sarcoma of the distal leg, who received additional excision after inadequate resection at the initial hospital (case 3), showed partial necrosis of the free vascularized latissimus dorsi flap and required secondary minor suturing.

Local recurrence was observed at the region outside of the irradiated field in the desmoid patient (case 1). Case 2 incidentally underwent an incisional biopsy of the calcaneal tendon at 61 months after the operation because she hoped to have cosmetic surgery to improve the appearance of operation site. A pathological examination showed that the patient was recurrence-free and also demonstrated the regeneration of the tenocytes of the irradiated calcaneal tendon. None of sarcoma patients had local recurrence or distant metastasis.

Cases 1 and 5 showed post-operative restricted ROM of the wrist joints; however, the restriction was mild (RTOG criteria: grade 1). No cases of neuropathy, bone necrosis, vascular obstruction, and lymphatic edema were observed.

The mean limb function scores, which were determined according to the MSTS scoring system, were 87 % in the patients with a tumor of the upper extremities and 100 % in the patients with a tumor of the lower extremities.

## Discussion

The surgical excision of sarcomas involving the tendons sometimes leads to severe functional disability [1, 4–6]. We showed the successful clinical outcomes of minimally invasive surgery using IOERT for extremity STS involving the tendon. Although the current study is only a pilot study with a small number of patients, the results show that the procedure has the potential to become a standard minimally invasive therapy for selected patients.

IOERT has several advantages over traditional EBRT. It allows the radiotherapist to create a highly conformal treatment field. It also allows for the delivery of higher doses than EBRT because it is less toxic to the normal tissue [17, 23, 26, 28, 31–34]. In conventional IOERT, the electron beam is delivered (with the surrounding normal tissue shielded by a lead cone) to the affected site after the excision of the tumor with a marginal, intra-lesional, or wide margin. The most important point of the current study is that the IOERT was applied to salvage the tendon prior to the resection of the peri-/intra-tendinous tumor. In the current study, IOERT was delivered with a wide margin, and the surrounding tissue was completely shielded with a lead board and applicator; the tumor was then excised with a marginal or intra-lesional margin. Thus, the current surgical procedure should be distinguished from conventional IOERT, which is applied after the resection of the tumor with an aim of reducing local recurrence.

There are few reports on the use of IORT as a minimally invasive treatment of the affected limb in patients with localized sarcoma. A group at Kyoto University first reported the application of IORT for extremity osteosarcoma [35, 36]. The lesion was isolated and separated from the normal tissues with a wide margin in the axial plane, maintaining the continuity of the proximal and distal normal tissue, and irradiation (45–70 Gy) was administered with an electron beam (12–30 MeV) or X-rays (10 MV). The irradiated tumor was generally left without excision. Twelve of 33 patients showed post-operative local recurrence. However, only one of the 12 patients showed recurrence in the irradiated field. The concept of the clinical study performed at Kyoto University was identical to our current study.

The biological effectiveness of delivering a dose in a single fraction is not completely understood. The tool most commonly used for the quantitative prediction of dose/fractionation dependencies in radiotherapy is the mechanistically based LQ model [29]. Using the LQ model and assuming an alpha/beta of three for the normal soft tissue surrounding a tumor, a single dose of 25 Gy corresponded to an EQD2 of 140 Gy. None of the sarcoma patients had local recurrences or distant metastasis. In case 2, the examination of a biopsy sample confirmed the absence of recurrence. Thus, 35–50 Gy might have been a radical dose in the current series. Further study and a long-term follow-up period are warranted.

The IOERT dose was carefully determined, so that it would achieve a biological cure while minimizing the damage to the soft tissue, such as the muscle, tendons, and peripheral nerves [37–40]. The adverse effects on the muscle were most critical, since IOERT was not applied to lesions that included a major nerve (such as the sciatic nerve or brachial plexus) in the current series. Radiation-induced muscle injury was characterized by a loss of muscle fiber, decreased fiber size, severe vessel lesions, hemorrhage, inflammation, coagulation necrosis, and fibrosis [37, 39]. In the context of clinical studies, IOERT is usually combined with EBRT with a dose range of 10–12 Gy for R0-R1 resections and 15–20 Gy for R2 resections (gross macroscopic residual disease) [37–40]. In the current series, case 5 showed mildly restricted ROM of the wrist joints. Because this patient underwent IOERT during additional wide excision after unplanned tumor resection, the flexor muscle of the distal forearm was included in the irradiation field to avoid recurrence in the contaminated area. However, the radiation-induced muscle injury did not cause any significant functional disability even in case 5, because the tumors were located at the distal extremities, which include only a small amount of muscle fiber. Furthermore, the muscle fiber included in the irradiation field was resected as much as possible. These findings suggest that this surgical procedure should be indicated only for tumors located in the distal extremities.

In our institute, it was necessary to transfer the patient from the operation room to the radiation treatment room. This process requires close cooperation between nursing staff, anesthesiologists, surgeons, and radiation oncology staff. To reduce the complications of IOERT, lumbar anesthesia/scalene block anesthesia was administered until the patients returned from the radiation treatment room (with the exception of one pediatric patient; case 1). The radiation oncologist and surgeons could therefore maintain direct visual and video contact with the patient. To ensure the smooth performance of this complicated procedure, it is recommended that it be thoroughly discussed with the anesthesiologist prior to surgery.

In one patient (case 1), local recurrence was observed outside of the irradiated field. This patient had a desmoid tumor which recurred three times after resection. Thus, the recurrence of this case was not due to the current surgical procedure; rather, it was due the pathological character of the desmoid tumor. Case 1 showed the restricted ROM of the wrist joints after surgery due to repeated surgery for desmoid tumors. The most critical problem associated with conventional EBRT is that it often causes a number of common, well-described side effects, such as severe muscle fibrosis, neuropathy, joint contracture, vascular obstruction, and lymphatic edema [14]. These adverse effects of EBRT sometimes compromise the quality of life of patients. However, in the current series of patients who underwent IOERT, no severe complications were observed. A long-term follow-up study is warranted.

Because the margin status is reported to be the most important prognostic factor for the local control of STS, the elevation of the tumor mass with a completely negative margin from the surrounding tissue is necessary before IOERT is administered. Thus, this surgical procedure must be carefully indicated for selected patients.

## Conclusions

When an STS involving a tendon is located at the distal part of an extremity, a wide excision usually results in severe functional disability. We therefore developed a minimally invasive surgical technique using IOERT to reduce the incidence of post-operative functional disability in patients with peri-/intra-tendinous STS. IOERT was delivered with a wide margin under the complete shield of the surrounding tissue using a lead board and applicator. The tumor was then excised with a marginal or intra-lesional margin maintaining the tendon continuity. We showed the successful clinical outcomes of this minimally invasive surgery.

Although the current study is only a pilot study with a small number of patients, it has the potential to become a standard therapy for selected patients. A further study with a long-term follow-up period is necessary.

## Abbreviations

EBRT, external-beam radiation therapy; IOERT, intraoperative electron-beam radiotherapy; IORT, intraoperative radiotherapy; MSTS, Musculoskeletal Tumor Society; ROM, range of motion; RTOG, Radiation Therapy Oncology Group; STS, soft tissue sarcoma
